# Editorial: Current status of and future directions for assessing technology acceptance for digital (mental) health interventions

**DOI:** 10.3389/fdgth.2024.1467297

**Published:** 2024-08-20

**Authors:** Jennifer Apolinário-Hagen, Giulia Paganin, Silvia Simbula

**Affiliations:** ^1^ Institute of Occupational, Social and Environmental Medicine, Centre for Health and Society, Medical Faculty, Heinrich-Heine University Düsseldorf, Düsseldorf, Germany; ^2^ Department of Education Studies “Giovanni Maria Bertin”, University of Bologna, Bologna, Italy; ^3^ Department of Psychology, University of Milano-Bicocca, Milan, Italy; ^4^ Bicocca Center for Applied Psychology, University of Milano-Bicocca, Milan, Italy

**Keywords:** telemedicine, UTAUT (unified theory of acceptance and use of technology), digital health interventions, digital mental health, digital mental health assessment and interventions, innovation diffusion, user perspective

**Editorial on the Research Topic**
Current status of and future directions for assessing technology acceptance for digital (mental) health interventions

Digital mental health interventions (DMHIs) have become increasingly widespread over the last twenty years as they demonstrated promise in the prevention and treatment of common mental health issues in a variety of settings. However, adoption is still low in many countries despite policy-makerś efforts, such as approving digital therapeutics (DTx). Developing acceptance-facilitating interventions (AFIs) and customizing DMHIs to user needs depend on an in-depth understanding of individual innovation acceptance. In fact, a growing number of studies considered measuring user acceptance and its determinants as well as attitudes and preferences among key stakeholders prior the utilization of digital health services.

The goal of this Research Topic is to gather and present empirical studies on the state of technology acceptance research dedicated to theoretical frameworks such as the Unified Theory of Acceptance and Use of Technology (UTAUT) as well as future directions for user-centered DMHIs. These assessments not only include acceptance of interventions improving mental health across various application fields and populations but also perspectives regarding therapeutic relationships and human-computer interactions.

This editorial article outlines nine contributions collected for this special issue and their role in enhancing our understanding of technology acceptance. Grounded on both quantitative and qualitative research methods, the results revealed a complex picture of the acceptance of digital interventions by different target populations.

According to the survey by Kählke et al., university students clearly favored face-to-face treatment over both stand-alone and blended DMHIs, while they highlighted a moderate acceptance for DMHIs. Reporting a mental illness, believing in DMHIś efficacy, and not intending to use traditional services were linked to a preference for DMHIs.

Based on the UTAUT model, Staeck et al. demonstrated that two latent classes of psychotherapists in training may be distinguished according to the model determinants, namely Performance Expectancy and Effort Expectancy. Interestingly, these classes also differed in therapeutic orientation.

Mental health professionals' attitudes and concerns regarding mobile health were investigated by Dominiak et al. Prioritizing telepsychiatry was indicated by the majority of them, with a surge in interest during the COVID-19 pandemic. A quarter of them expressed concerns like challenges in precisely evaluating patients' conditions and technological issues.

This Research Topic comprised two randomized controlled trials (RCTs) on AFIs. Knauer et al., building on the UTAUT, examined the acceptability of smart sensing, acceptance determinants, and the efficacy of a video-based AFI in comparison to a mindfulness video. At baseline, smart sensing was moderately accepted. Acceptance was found to be determined by trust, social influence, and performance expectations. The AFI, however, had no significant influence on acceptance ratings.

Another UTAUT-based RCT was conducted by Rottstädt et al. on promoting smart sensing's adoption. In contrast to an active control group, the AFI consisted of showing a smart sensing video. Acceptance increased moderately in the intervention group. The main factors that determine acceptance were found to be Performance and Effort Expectancy.

Lastly, this Research Topic included four qualitative studies.

A focused ethnography on the implementation, acceptance, and use of modern nursing technologies was carried out by Klawunn et al. The authors discovered that a product's acceptance or rejection does not always correspond to its use. Users’ approval of technology before it is implemented frequently takes the form of prejudice, but after they have some time to test it, their intention to utilize it can turn to sustained use.

The interview study by Posselt et al. on patients' attitudes towards and intention to use DTx for depressive disorders indicated that patients do not view apps on prescription as a replacement for face-to-face treatment in terms of performance expectancies. While general practitioners play a vital role through prescriptions, effort expectations encompassed both possible benefits and obstacles linked to technical, motivational, and skill-related components.

The qualitative study by Carlisle et al. examined online forums in an effort to help young people in rural areas become more resilient. Their findings indicated that online peer support forums help strengthen resilience and a sense of belonging, as they provide a virtual space for social connections, to share information, gain knowledge, and offer mutual support.

Finally, the qualitative study by Abi Ramia et al. investigated the feasibility and uptake of Step-by-Step (SbS), a DMHI for depression. Their results revealed high acceptability of SbS among users, but it also identified subgroups for which acceptance or use might be lower, such as older users and those with restricted access to the internet or smartphones.

Taken together, recent research shows that face-to-face interactions are still favored, whereas attitudes regarding DMHIs are becoming more positive. Innovation diffusion takes time and is context-sensitive. Acceptance appears higher among those who have already dealt with mental health issues or who believe in the added value. Besides structured programs, easily accessible online forums can promote mental health by providing peer support in young people. Professionals appear largely supportive of DMHIs, which have gained acceptance throughout the COVID-19 pandemic, although they are worried about various barriers.

Consequently, promoting the informed use of DMHIs requires the active participation and education of health professionals. In line with prior research, it was found that UTAUT determinants, particularly performance expectancies, alongside with other factors like trust, represent drivers of user acceptance. Under certain conditions, AFIs such as educational videos could increase the acceptability of DMHIs. Research also emphasizes the need to differentiate between early adoption and continued use, as well as the unique needs of different populations, varying in demographics and preferences.

As digital health continues to change the landscape of health-promoting settings, it remains important to comprehend how new technologies are viewed in order to assist their uptake. Gaining insight into the factors that influence the uptake and effectiveness of digital interventions could thus help reduce the gap between the demand and supply for personalized DMHIs while improving the access to both in-person and digital interventions. Expanding the scope of research beyond the UTAUT is essential for designing and disseminating user-centered interventions, especially in light of the interaction of individual, organizational, and environmental factors in technology acceptance.

